# A critical review on microbial carbonate precipitation via denitrification process in building materials

**DOI:** 10.1080/21655979.2021.1979862

**Published:** 2021-10-15

**Authors:** Surabhi Jain, Chaolin Fang, Varenyam Achal

**Affiliations:** a Environmental Science and Engineering Program, Guangdong Technion – Israel Institute of Technology, Shantou, China; b Department of Civil and Environmental Engineering, Technion – Israel Institute of Technology, Haifa, Israel

**Keywords:** Biomineralization, denitrification, building materials, soil reinforcement, up-scaling

## Abstract

The naturally occurring biomineralization or microbially induced calcium carbonate (MICP) precipitation is gaining huge attention due to its widespread application in various fields of engineering. Microbial denitrification is one of the feasible metabolic pathways, in which the denitrifying microbes lead to precipitation of carbonate biomineral by their basic enzymatic and metabolic activities. This review article explains all the metabolic pathways and their mechanism involved in the MICP process in detail along with the benefits of using denitrification over other pathways during MICP implementation. The potential application of denitrification in building materials pertaining to soil reinforcement, bioconcrete, restoration of heritage structures and mitigating the soil pollution has been reviewed by addressing the finding and limitation of MICP treatment. This manuscript further sheds light on the challenges faced during upscaling, real field implementation and the need for future research in this path. The review concludes that although MICP via denitrification is an promising technique to employ it in building materials, a vast interdisciplinary research is still needed for the successful commercialization of this technique.

## 1. State of the art of microbial carbonate precipitation

The evolution and growth of the amazing nature is maintained with the involvement of various physiochemical and/or biochemical reactions or phenomena. Exploring, exploiting or mimicking nature’s biochemical phenomenon for various engineering applications is known as ‘nature-inspired innovation’. Some remarkable examples of nature-inspired innovation are the wing clap of butterfly explaining the flying mechanism of flights, the self-cleaning mechanism of lotus leaves giving formula for different industrial products, the biomineralization process of rock formation applied to form new minerals for different applications and so on [1, 2–4, 5].

Among all of the biochemical process, the concept of biomineralization is gaining huge attention among various engineers and researchers due to its widespread application in the field of environmental, chemical, biological, and building materials, earth science, and geotechnical engineering [3,6–8]. Minerals formed by biological activities of organisms or plants and/or by their byproduct is known as biomineralization. The biomineral formation is a ubiquitous phenomenon due to the participation of nearly all the taxonomic group of biological kingdoms. Biominerals can be biologically controlled, influenced or induced mineralization pertaining to their mechanism of formation.

Until now, nearly sixty-four varieties of minerals such as carbonates, silicates, phosphorites, iron and manganese oxides, and sulfide minerals have been discovered, which are formed via biologically induced or controlled mineralization processes [9]. Also, researchers are trying to explore the precipitation of minerals other than the existing sixty-four varieties by different biological activities. Among different mineral formation, the microbially induced carbonate precipitation (MICP) is abundant due to its occurrence in various adverse and extreme soil, marine, terrestrial and aquatic conditions with a wide variety of organisms and plants involved [10,11]. Several metabolic pathways of both prokaryotic and eukaryotic microbes are involved in the microbially induced carbonate precipitation. Among various pathways, MICP via ureolysis or urea degradation has been extensively studied pertaining to isolation of ureolytic microbes in different and extreme locations, utilization of *S. pasteurii* as a standard organism, effect of prevailing biochemical and environmental conditions on ureolysis and MICP, employing augmentation or stimulation or biogrouting for various applications, etc. [12–16]. Various laboratory studies and field trials have shown the application of ureolysis-driven MICP in soil strengthening, remediation of heavy metals and radionuclides, building materials, enhanced oil recovery, CO2 sequestration, etc. since 2000 [14,17].

Although highly capable ureolytic microbes have been isolated from different locations, these are not ubiquitous in nature [18]. Therefore, bioaugmentation, i.e., introduction of ex-situ ureolytic microbe technique has to be employed under various harsh conditions such as high pressure, highly acidic or alkaline, high concentration of salt, low moisture and nutrient conditions. Employing the augmentation process is not viable compared to stimulation because of high cost, disturbance to the surrounding, adverse effect of the environment on ureolytic microbes and vice versa. The major problem associated with MICP via ureolysis is the hindrance of microbial activity in low or the absence of oxygen due to the aerobic nature of ureolytic microbes [1,19–20]. Hence, the ureolytic activity and mineral precipitation get hindered in most of the MICP application processes such as in oil reservoirs, healing the concrete crack, subsurface soil reinforcement, soil remediation below ground water, etc. due to the lack of oxygen. Also, the generation of ammonia as an end product is a concern for its toxicity to soil, plants and water body and extra effort and cost are involved to remove or reutilize the ammonia [21,22]. Therefore, the other metabolic kinetics involved in MICP need to be considered, compared and comprehended to evaluate the most feasible alternative pathways pertaining to MICP application. Also, some researchers have conducted experiments on denitrification-based carbonate precipitation for an efficient MICP application in recent times [23–25].

In view of this, this manuscript presents a critical review on the detailed mechanism of all the pathways involved in MICP and the feasibility of these pathways in MICP-driven application. The cause of research on denitrification and its potential over other pathways for sustainable MICP application is evaluated. This manuscript further reviewed the application of denitrification-based MICP in building materials such as stabilization of geomaterials, mitigating the soil pollution, restoring the heritage structure and self-healing concrete in detail. The upcoming challenges for upscaling the process, future possibility and required research to implement it in real field are presented. Furthermore, the sustainability in terms of durability, cost feasibility and viability of denitrification and its effect on sustainable land use planning, management and sustainable construction industry activities are also proposed.

## 2. Mechanism of MICP via various microbial metabolic pathways

Microorganisms are present in almost every habitat on earth [26]. Both heterotrophic and autotrophic microorganisms undergo various metabolic pathways and increase the total carbonate (CO_3_^2-^) content and pH of the system, resulting in the precipitation of carbonate biomineral. The autotrophic pathways such as photosynthesis and methane oxidation and heterotrophic pathways such as nitrogen and sulfur cycle and its pros and cons for MICP application are discussed in detail in the following section.

### 2.1 Autotrophic pathways

#### 2.1.1 Photosynthesis

In the photosynthetic process, the alkalinity across the microbial cell increases during the exchange of HCO_3_^−^/OH^−^ ions. Here, the microbes utilize gaseous or dissolved CO_2_ to form organic matter via photosynthesis. Simultaneously, bicarbonate is converted into CO_2_ and OH^−^, eventually forming carbonate mineral [10]. The photosynthetic microbes mainly responsible for carbonate mineral precipitations are cyanobacteria, purple photosynthetic bacteria and microalgae. Nearly 70% of carbonate rocks in earth were formed due to cyanobacteria [27]. Different forms of carbonate minerals were found in diverse environments such as freshwater, marine water, hot springs and terrestrial areas, in which most are formed via the microbial photosynthesis process [18,28–30]. However, applying this process for engineering or building material application is still a question because of the need of constant sunlight and inorganic carbon during photosynthesis and carbonate biomineral precipitation [31].

#### 2.1.2 Methane oxidation

Methane is the second greenhouse gas that can be captured using various bioengineering processes [32]. Under aerobic or anoxic conditions, the methane gets oxidized to methanol, which further forms formate by microbial enzymatic activity. The formate equilibrates and produces formic acid, carbon dioxide and hydroxyl ion, leading to the rise in the system alkalinity. Finally, carbonate minerals are formed by the generation of carbonate ions from carbon dioxide [22,33]. These aerobic methane oxidation processes are described in the equation form (Equations 1 to 3). In the anaerobic methane oxidation process, the bicarbonate ions and carbonate mineral are formed by utilizing sulfate as an electron acceptor instead of oxygen (Equations 4 and 5),

(1) $$CH_{4}+O_{2}\to HCOOH+OH^{-}+H_{2}O,$$

(2) $$HCOOH\to CO_{2},$$

(3) $$M^{2+}+CO_{2}+2OH^{-}\to MCO_{3}+H_{2}O,$$

(4) $$CH_{3}^{2-}+H_{2}O↔HCO_{3}^{-},$$

(5) $$CH_{4}+SO_{4}^{2-}\to HCO_{3}^{-}+HS^{-}+H_{2}O.$$

**Figure 1.**
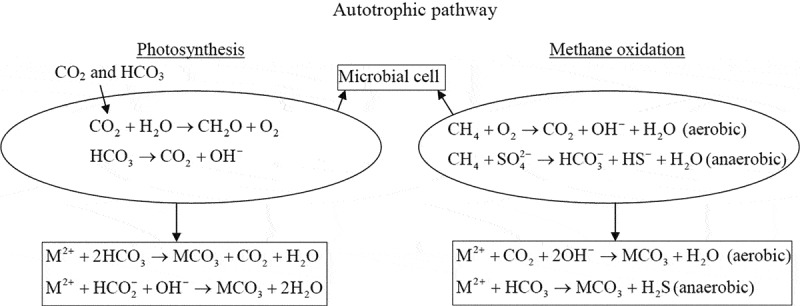
Photosynthesis and methane oxidation mechanism for carbonate precipitation around the microbial cell

Few anaerobic methane-oxidizing microorganisms have been isolated from diverse environments such as Kidd mud volcano in the gulf, salt dome cap rocks and Tuscan archipelago and the mechanism of bicarbonate formation via methane oxidation was evaluated [34, 35, 36]. Also, as reported in other studies, *Methylo cystisparvus* microbe was employed and optimized for the calcium carbonate biomineral formation to produce environment-friendly building materials via the methane oxidation mineralization process [37–39]. Although this metabolic pathway is well-described and carried out under laboratory conditions, implementing it in prevailing in situ environmental conditions demands further research and analysis. Figure 1 describes both photosynthesis and methane oxidation mechanisms for carbonate precipitation around the microbial cell.

### 2.2 Heterotrophic pathways

In general, heterotrophic microbes utilize various organic carbon sources and produce different metabolic end products in the form of CO_2._ The water and oxidized CO_2_ hydrolyze to form carbonic acid, which assists the carbonate mineral precipitation. The heterotrophic pathways involved in MICP such as sulfur cycle and nitrogen cycle are discussed below.

#### 2.2.1 Sulfur cycle

The reduction of sulfate occurs via sulfate-reducing bacteria producing bicarbonate ions and hydrogen sulfide under anoxic and anaerobic conditions. The generation of biocarbonate and carbonate ions assists carbonate biomineral formation. However, it mainly depends on the behavior of hydrogen sulfide since it affects the pH of the system. For instance, the degasing of H_2_S and oxidation of sulfide to sulfur by anoxygenic sulfide phototrophic bacteria lead to raise in the system pH and subsequently favor the intra- or extracellular biomineral formation [18]. On the contrary, H_2_S can be oxidized to sulfate ions and produces sulfuric acid by autotrophic aerobic sulfide-oxidizing bacteria. Sulfuric acid decreases the pH and inhibits the biomineral precipitation. The biomineralization or MICP via the sulfur cycle is not feasible for engineering application because incessant maintaining of the anaerobic condition is quite difficult under real-field conditions. Also, the odorous hydrogen sulfide gas is highly toxic to the environment.

#### 2.2.2 Nitrogen cycle

The nitrogen cycle involves three different metabolic pathways, i.e., amino acid ammonification, nitrate reduction, and urea degradation, which can favor the carbonate-based biomineral formation.

In the amino acid ammonification, CO_2_ and ammonia are generated by the microbial metabolic process. The ammonia gets hydrolyzed to ammonium and hydroxide ions, as shown in equations 6 and 7. The hydroxide ion increases the system pH and the formation of bicarbonate ions by CO_2_ favors the precipitation of carbonate biomineral. The aerobiosis species such as *Myxococcus xanthus* and *Alcanivorax borkumensis* are abundantly present in all environments and use amino acids as their sole energy source [40–42]. In this process, the microbes serve as a nucleation site and form different polymorphs of calcium carbonate in the presence of free divalent calcium ions [43,44],

(6) $$Acid+O_{2}\to NH_{3}+CO_{2}+H_{2}O,$$

(7) $$NH_{3}+H_{2}O\to NH_{4}+OH^{-}+OH^{-},$$

(8) $$CO_{2}+OH^{-}\to HCO_{3}^{-},$$

(9) $$HCO_{3}^{-}+M^{2+}\to MCO_{3}+H^{+}.$$

#### 2.2.3 Urea degradation or ureolysis

In the urea hydrolysis process, the urea is hydrolyzed by microbial urease enzyme and generates ammonia and carbamic acid. Carbamic acid further hydrolyzes and produces ammonia and carbonic acid [45]. These products further get hydrolyzed to generate bicarbonate ions, as shown in below equations (Equation10-12). The formation of ammonium ions and hydroxyl ions due to the reaction between ammonia and water increases the alkalinity of the surrounding environment (Equation 13). Under the high alkaline condition, the over saturation of divalent cations hastens the biocarbonate mineral formation. During urea hydrolysis, the given urea also acts as a major nitrogen source for the various microbial species [46],

(10) $$CO(NH_{2}{)}_{2}+H_{2}O\overset{urease}{⟶}NH_{2}COOH+NH_{3},$$

(11) $$NH_{2}COOH+H_{2}O\to NH_{3}+H_{2}CO_{3},$$

(12) $$H_{2}CO_{3}\to 2H^{+}+2CO_{3}^{2-},$$

(13) $$NH_{3}+H_{2}O\to NH^{4+}+OH^{-},$$

(14) $$Ca^{2+}+2CO_{3}^{2-}\to CaCO_{3}.$$

Several ureolytic microbial strains such as *B. megaterium, B. subtilis, P. Vulgaris, B. sphaericus, B. thuringiensis, Sporosarcina pasteurii, S. ginsengisoli, Kocuria flava*, and species of *Sporolactobacillus* have been isolated to utilize the MICP in building materials and other engineering applications [7,47–54]. Among all the microbes isolated, the Gram-positive, aerobic, and rod-shaped *Sporosarcina pasteurii* was found to have maximum ureolytic activity and higher mineral precipitation rate and to be most utilized bacteria until now [55–58]. The studies showed that at the pH of 9, *S. pasteurii* can induce 98% of precipitation against only 54% attained during the chemical process, under similar environmental conditions [59,60].

Although numerous laboratory and limited MICP studies via the ureolytic process have shown promising results, the end products, i.e., ammonia and ammonium, are undesirable and potentially toxic to the ecosystem. A small quantity of ammonium can be converted to nitrate and further to nitrogen via nitrification and denitrification. But the quantity of ammonium generation and its complete utilization by the nitrifying bacteria is still a question [61–63]. MICP via microbial ureolysis can be feasible for specific applications only if the end products can be further utilized such as ammonium chloride as in situ fertilizer [64].

#### 2.2.4 Nitrate reduction

In nature, the denitrifying microbes balance the dinitrogen amount of atmosphere by reducing the terrestrial nitrate to dinitrogen gas. Also, the presence of denitrifying microbes in specific locations such as landfill, polluted areas, and eutrophic lakes is beneficial to the ecosystem due to the removal of nitrogen via denitrification.

In the denitrification process, denitrification of nitrate occurs in the presence of organic matter to generate alkalinity, carbon dioxide and nitrogen gas [65]. Furthermore, carbon dioxide equilibrates with water and forms biocarbonate ions. In this alkaline environment and in the presence of divalent cations, carbonate biomineral forms [18]. The denitrification process is explained in the below equations (Equations 15–17). The denitrifying microorganism such as *Pseudomonas denitrificans, Alcaligenes, Denitro bacillus, Thiobacillus, Spirillum* and *Micrococcus* are typically facultative anaerobes and usually present in the subsurface environment [66],

(15) $$(CH_{3}COOH{)}_{2}+NO_{3}^{-}\to \frac{4}{5}N_{2}+3CO_{2}+3H_{2}O+OH.$$

(16) $$3CO_{2}+3H_{2}O\to HCO_{3}^{-}+H^{+}.$$

(17) $$M^{2+}+HCO_{3}^{-}+OH^{-}\to MCO_{3}+H_{2}O.$$

**Figure 2.**
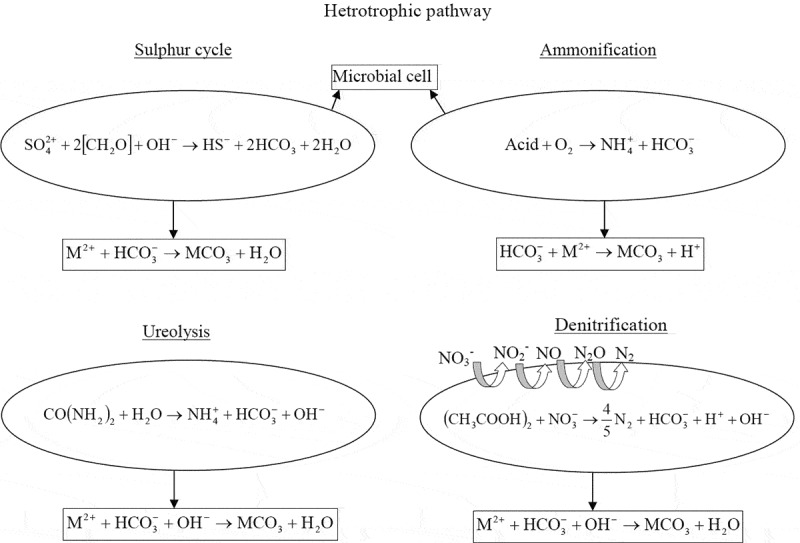
Mechanism of heterotrophic pathways for carbonate biomineral precipitation

This biologically induced denitrification process occurs through multiple reactions, which are carried out by different enzymatic processes. Although the byproduct N_2_ is less harmful, the intermediate product during MICP denitrification, i.e., nitrite and nitrous oxide, is detrimental to the environment. Figure 2 depicts all the heterotrophic pathways involved in the MICP process.

In all the above reactions, M^2+^ signifies the cation present and MCO_3_ is the carbonate biomineral formed. Various biominerals such as different polymorphs of calcium carbonate (calcite, vaterite, and aragonite), dolomite (CaMg(CO_3_)_2_), magnesite (MgCO_3_), strontianite (SrCO_3_), rhodochrosite (Mn, Fe, Mg,CaCO_3_), etc. are formed via the MICP process [9,67,68]. In the absence of divalent cations, bicarbonate and carbonate ions accumulate in the alkaline environment due to microbial activity, causing zeolite crystals to form. Soda lakes are examples of the formation of zeolite due to various metabolic activities [69]. In earth, more than 50% of known carbonate minerals are calcium carbonate due to the presence of calcium ions in most of the soil or aquatic system and this ion takes part in most of the cellular metabolism processes and in microbial cell functions [3,67,70]. Therefore, the microbially induced carbonate precipitation is often known as microbially induced calcium carbonate precipitation. For the same reason, until 1980, the term calcification was in use for the biomineralization term.

In all the metabolic pathways for MICP, microbes not only help by their metabolic activity but also serve as nucleation templates for the new mineral formation [68]. Bacterial cell walls are mostly negatively charged surfaces with the presence of carboxyl and phosphate groups [71–73]. The negatively charged functional groups adsorb the available cations such as Ca^2+^, Mg^2+^, Fe^3+^, Cu^2+^, Mn^2+^, and Zn^2+^ from the system and initiate the mineral precipitation by acting as a nucleation site [18,74]. Due to the microbial nucleation, the energy barrier for biomineral formation is reduced and the rate is faster than chemical precipitation [75,76]. Interestingly, even the metabolically inactive bacteria such as *D. desulfuricans* also serve as heterogeneous nucleation sites during calcium carbonate precipitation [77,78]. Apart from the microbial cell surface, the EPS formed around the microbial cell also serves as heterogeneous nucleation sites for mineral precipitation [22]. However, sometimes, EPS can inhibit the precipitation by trapping and reducing the saturation of divalent cations in the system [79,80].

Although the above-mentioned metabolic pathways lead to mineralization in terms of carbonate precipitation, all are not equally feasible for engineering application. The following section presents some essential factors that need to be considered to employ any pathway for engineering application and compared them to choose the best alternative metabolic pathway to apply in sustainable building materials.

## 3. Evaluation of all metabolic pathways for efficient MICP application

Literature listed four major criteria, i.e., solubility of cementation reagents, reagent requirement, rate of biomineral formation and generation of secondary products to demonstrate the efficiency of the different metabolic pathways on soil reinforcement via MICP [81]. The other important criteria need to be considered for sustainable MICP application in building materials are availability of microbes, nature of microbes in terms of aerobic or anaerobic growth, and cost associated in employing it in field. The above-mentioned factors are analyzed in detail for every metabolic pathway in this section. Table 1 presents the theoretical evaluation of all the autotrophic and heterotrophic pathways by considering the above-mentioned factors.

**Table 1.** Evaluation of different metabolic pathways for efficient MICP application in building materials * **∏** symbol is for okay and × symbol is for not okay on the mentioned factor

Factors	Metabolic pathways					
	Autotrophic		Heterotrophic			
	Photosynthesis	Methane oxidation	Sulfur cycle	Ammonification	Urea hydrolysis	Nitrate reduction
**Solubility**	**×**	**×**	**×**	**×**	**∏**	**∏**
**Requirement of reagents**	**××**	**××**	**××**	**××**	**××**	**∏∏**
**Rate of precipitation**	**××∏**	**××∏**	**××∏**	**××∏**	**××∏**	**∏∏∏**
**Availability of microbes**	**××∏∏**	**××∏∏**	**××∏∏**	**××∏∏**	**××∏×**	**∏∏∏∏**
**Nature of microbes**	**××∏∏×**	**××∏∏∏**	**××∏∏×**	**××∏∏×**	**××∏××**	**∏∏∏∏∏**
**Intermediate product**	**××∏∏×∏**	**××∏∏∏∏**	**××∏∏×∏**	**××∏∏×∏**	**××∏××∏**	**∏∏∏∏∏×**
**End product**	**××∏∏×∏×**	**××∏∏∏∏×**	**××∏∏×∏×**	**××∏∏×∏×**	**××∏××∏×**	**∏∏∏∏∏×∏**
**Cost**	**××∏∏×∏××**	**××∏∏∏∏××**	**××∏∏×∏××**	**××∏∏×∏××**	**××∏××∏××**	**∏∏∏∏∏×∏∏**
**Total**	**××∏∏×∏××**	**××∏∏∏∏××**	**××∏∏×∏××**	**××∏∏×∏××**	**××∏××∏××**	**∏∏∏∏∏×∏∏**

Pertaining to solubility, chemical reagents utilized for ureolysis is most soluble compared to other metabolic pathways. Sulfate reduction and ammonification are not at all viable for building materials due to its poor solubility of reagents. However, during denitrification, the solubility of substrate with calcium makes it feasible for MICP application but less than ureolysis [81]. The autotrophic pathways need constant sunlight and/or inorganic carbon makes it not feasible to implement it in MICP-driven sustainable building materials such as soil reinforcement or remediation in high depth. The literature concludes that all the heterotrophic pathways need almost an equal quantity of substrates for one mole of biomineral production except ammonification [81]. But, in the denitrification process, 1 mole of acetate generate 2 moles of CO_2_, which further generates more carbonate ions compared to only one mole of HCO_3_^−^ generation per mole of urea in ureolysis. It concludes that compared to ureolysis, denitrification requires lower substrate concentrations for an equal amount of carbonate biomineral production [18,82]. If calcium acetate (Ca(CH_3_CO_2_)_2_) is provided, it serves as both an electron donor and a calcium ion provider. It helps to minimize the cost as well as potential environmental impacts by eliminating the extra addition of both CaCl_2_ for calcium ions or chemicals for electron donors [83]. Denitrification can occur at a low concentration of nitrate (NO_3_^−^), for example, *Pseudomonas* species can easily grow in 0.080 mM of NO_3_^−^ concentration [84–87]. Therefore, the denitrifying microbes do not require exogenous nitrate content during denitrification for MICP application. However, a higher concentration (>25 mM) of nitrate can inhibit the growth and microbial activity of denitrifying microbes leads to no mineral precipitation [66]. The adequate nitrate amount required for denitrification needs to be optimized for an efficient MICP process under real field conditions.

Pertaining to rate, the rate of biomineral precipitation mainly depends on the biomass concentration, its growth rate and microbial enzymatic activity involved. Also, the rate of precipitation can be manipulated by altering the enzymatic activity, biochemical concentration, temperature, etc. as per the engineers’ specific requirements [88,89]. For instance, the slower rate of precipitation freely allows the transport of biomass and chemical reagents to a larger depth, which is beneficial for subsurface MICP application such as biogrouting for stabilization, bioclogging for modifying the hydraulic conductivity, etc. On the other hand, a higher precipitation is required for instant healing of the crack of the soil zone or concrete. It is essential to search the suitable native microbes or introduce ex situ microbes in the intended zone to implement any pathways via bioaugmentation or biostimulation. However, as discussed, biostimulation is more feasible than the augmentation process for MICP application in building materials. Compared to all other metabolic pathways, the denitrifying microbes utilized for nitrate reduction are ubiquitous in the soil surface, subsurface and the aquatic zone [66,84,87,90]. Hence, biostimulation without any introduction of foreign microbes can be effortlessly applicable under the prevailing site condition for MICP treatment with low cost [65]. Also, the effect of the prevailing environmental condition on microbial activity and vice versa will not be a matter of concern during denitrification based MICP due to utilization of indigenous microbes in field.

The application of MICP in building materials mostly occurs in low oxygen, i.e., anoxic or anaerobic conditions such as deep soil subsurface, oil reservoirs, inside concrete or brick structures. Therefore, the utilized microbial growth and activity should not get inhibited in the absence of oxygen, which means that it should be anaerobic in nature. However, the obligate anaerobic nature of microbes can be problematic if there is a presence of oxygen (atmospheric concentration). It concludes that it is always preferable to use facultative anaerobic microbes than aerobic or anaerobic microbes. Previously, in the sulfur cycle, it was shown that the sulfur reduction assists the mineral precipitation only under strict anaerobic conditions, which makes this process not feasible for low oxygen or aerobic conditions. Similarly, the ammonification and urea hydrolysis occur mostly under aerobic conditions and get hindered in the absence of oxygen due to the inhibition of aerobic microbial growth under anaerobic conditions. Unlike the activity of ammonification and ureolytic bacteria, the metabolic activity of denitrifying bacteria is not affected by low or no oxygen conditions due to its facultative anaerobic nature [91]. Hence, it is perfectly possible to implement the denitrification process for subsurface soil treatment, oil recovery, and carbon sequestration (anoxic conditions) by providing adequate chemical reagents or electron donors. Literature studies suggested that the denitrification is also efficient in the well-aerated soils [206]. The other advantages of denitrification are having a higher degree of feasibility and more dominant mechanisms over other metabolic pathways due to its thermodynamic stability and higher standard Gibbs energy. The change in standard Gibbs energy for denitrification is more than an order of magnitude for ureolysis, −785 kJ/mol acetate and −27 kJ/mol acetate [92]. Methane oxidation-driven carbonate precipitation can also occur under both aerobic and anaerobic conditions but need further research to implement it in real field conditions due to the toxic hydrogen sulfide generation.

Table 2 shows the intermediate and final byproducts generated in different metabolic pathways involved in the MICP process. All the metabolic pathways have some undesirable intermediate or end byproduct generation, which is harmful to the ecosystem or environment such as hydrogen sulfide in the sulfur cycle and methane oxidation and formaldehyde during photosynthesis. As shown in the table, the advantage of denitrification over ureolysis and sulfate reduction for MICP precipitation is the nontoxic end product, i.e., N_2_ gas and small amount of carbon dioxide. In the literature, some of the previous studies also utilized the inert nitrogen gas to mitigate the liquefaction potential of geomaterial [93–96]. But the ammonia generated during ammonification and ureolysis is tightly bound in soil, whereas nitrate and nitrite are easily washed out and have an adverse effect on the quality of water. Also, the ammonia generated can be utilized in the fuel production and carbon capture [97–100]. The intermediate products generated during denitrification, i.e., nitric oxide (NO) and nitrogen dioxide (NO_2_), are harmful since they are potent greenhouse gas, major constituents of acid rain and the reason of destruction of the protective ozone layer [22,101,102]. However, for an efficient MICP, implementation via denitrification needs full completion of the multiple reaction by giving the end product as N_2_ gas and the biomineral. The last criterion is cost, which mainly depends on the cost of reagents and microbes, implementation methods such as augmentation or stimulation and removal of toxic products if generated. Keeping this in view, denitrification is a feasible alternative due to the low amount of substrate requirement, less harmful end product generation, and utilizing augmentation method due to the ubiquitous nature of microbes.

**Table 2.** Byproducts and its effect of different metabolic pathways involved in MICP

Metabolic pathways	Byproducts	Consequences
Photosynthesis	Oxygen (O_2_) and formaldehyde (CH_2_O)	Hazardous to health
Methane oxidation	Hydrogen sulfide (H_2_S)	Toxic, odorous gas
Sulfur cycle	Carbon dioxide and hydrogen sulfide (H_2_S)	Toxic, odorous gas
Ammonification	NH_3_ (ammonia)	Toxic gas
Urea hydrolysis/ureolysis	NH_3_ (ammonia) and NH_4_^+^ (ammonium)	Toxic gas Forms toxic salts
Denitrification	Nitric oxide (NO) and nitrogen dioxide (NO_2_) (intermediate) N_2_ and carbon dioxide (complete)	Intermediate products are detrimental for aquatic systems, agriculture and atmosphere

## 4. Potential applications in building materials via denitrificationbased MICP biotechnology

### 4.1 Stabilization of various geomaterials

**Figure 3.**
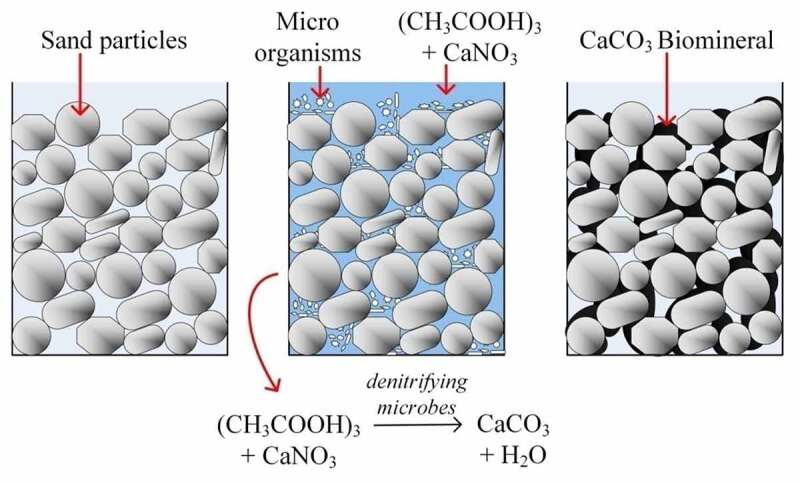
Schematic diagram of the biocementation process inside a soil matrix

Infrastructures are increasing unevenly with the increase in the population and urbanization. In most of the areas, the in situ mechanical and geotechnical properties such as strength, hydraulic conductivity, stiffness, compressibility, etc. of soil are not suitable for foundation and construction of roads, railways, dikes and different infrastructures. In this regard, biogrouting via MICP is gaining huge popularity because of being environmentally friendly, less energy requirement, and cost-effective method compared to conventional mechanical or chemical modification techniques. Biogrouting is a sustainable technique that can be applied in field without disturbing the nearby infrastructure [58]. In a soil phase system, the deposited biominerals provide cohesion to the soil particle by creating an effective bridge between the soil grains, which, in turn, modify the in situ geotechnical properties of geomaterial during MICP treatment [18,64]. Figure 3 depicts a schematic image of the concept of modifying the engineering properties of the sand column via the MICP process.

Different ureolytic and denitrifying microbes have been utilized to enhance the geomaterial properties via the MICP technique under laboratory conditions [6,56,103]. In 2008, the first study reported a weakly cemented sand column with a small quantity of carbonate biomineral precipitation via *P. denitrificans* within 40 days of MICP treatment [66]. A precipitation rate of 1.5 kg CaCO_3_ per m^3^ of soil in a single day was observed using isolated *Castellaniella denitrificans* [103]. It concludes that minimum sixty days is required to achieve 100 kg carbonate per m^3^ of soil for a minimal soil reinforcement. The study has shown that occurrence of intermediates waste and nonhomogeneous distribution of mineral over the length of soil column have a negative impact on the rate of enhancement. The study also suggested that a detailed investigation and optimization is essential for implementing denitrification as *in situ* soil reinforcement. Furthermore, studies have shown that denitrification is a promising technique for biomineral formation under anoxic and saline conditions, which further helps to modify the mechanical properties such as reduction of hydraulic conductivity of sand and coarse gravel and crack sealing of subsurface [65, 83 and 82, 104]. Furthermore, the MICP technique was also optimized by altering the injection strategy (number of flushes and residence time) and substrate concentrations [105]. It shows that the optimized MICP technique via denitrification has a higher rate of precipitation and soil enhancement than ureolysis. However, no field studies have been carried out pertaining to soil reinforcement via denitrification. Upscaling the study in big soil tanks or fields, monitoring the parameters during the process will give a better understanding about the feasibility of the technique.

Not only carbonate mineral precipitation but also the generation of nitrogen gas as a byproduct is also advantageous of utilizing the denitrification process. The generated biogas lessens the bulk stiffness of the pore fluid and hydraulic conductivity and pore pressure during cyclic or dynamic loading lead to an increase in the liquefaction resistance [106–108]. Hence, denitrification without any mineral precipitation can also be utilized as a potential soil reinforcement technique in terms of liquefaction mitigation [25,109]. In 2012, Rebata-Landa and Santamarina did the pioneering work to mitigate the liquefaction of soils by using *Paracoccus denitrificans* microbes. Furthermore, the studies revealed that a 80 to 95% reduction significantly affects the undrained stress-strain behavior, reduces the pore pressure and makes the sand non-liquefiable [93,94]. The microscopic studies of the gas bubble by the computed tomographic image revealed that the gas bubbles are present in small pockets of all the pores and bigger than the average grain size [110]. MICP via denitrification is more feasible if applied in the two-stage process for liquefaction mitigation. In the first stage, the biogas helps to desaturate the soil and strengthens it against the cyclic loading, which provides a temporary solution. In the second stage, a low amount, i.e., 1.5–2% of carbonate, binds the soil grains via biocementation and alters the mechanical properties of geomaterial, resulting in a long-term mitigation solution [95, 111].

In 2021, Mousavi and Ghayoomi examined the liquefaction potential of sands with various silt contents. The results concluded that a small degree of reduction in saturation, i.e., 4–5% of soil, did not liquefy regardless of fine content. In the study, the developed equation can predict the pore water pressure with different degrees of saturation in silty sands. To evaluate the MICP feasibility in natural soil, in Ref 112, the cyclic resistance experiment have been carried out in a triaxial setup by using a natural alluvial soil collected from Richmond, British Columbia, Canada. The results conclude that treatment is conducted by upward flow, whereas in field, the solutions or microbes are injected laterally, which have an impact on the rate of modification pertaining to soil permeability [104]. Also, unlike the standard sand, the natural soil is not homogeneous and consists of different layers or stratification, which further impact the pore size and gas trapping inside the pores and liquefaction potential. Furthermore, Ref 23 used a large tank to evaluate the MICP performance on a larger scale by monitoring the saturation, pore pressure, distribution of substrates and generated gas and its effect on pore pressure distribution and hydraulic conductivity. The study concludes that a single MICP treatment cycle is enough for a larger depth of soil treatment pertaining to liquefaction mitigation via the biostimulation process. However, the biogenic gas distribution, quantity and nature of biomineral, pore size and its effect on short-term and long-term hydraulic conductivity and other mechanical properties need to be studied further to implement it under in situ conditions [113].

### 4.2 Mitigation of soil pollution

Biologically induced mineralization has great potential for the immobilization of toxic metals and radionuclides from polluted soils by adsorption and/or complexation, redox reaction and precipitation and/or co-precipitation [14]. The conventional bio- or chemical remediation methods such as biosorption, accumulation, ion exchange, electrochemical treatment, etc. are unfeasible, reversible or extremely costly [20, 166,114b]. Compared to it, biomineralization or MICP-based remediation techniques are a stable, irreversible, eco-friendly and cost-effective remediation approaches [20, 1].

In MICP, the microbial metabolic activity raises the alkalinity of the system and produces carbon dioxide or bicarbonate ions, which facilitates the formation of carbonate ions. Simultaneously, in the system, due to the supersaturation of the divalent cation such as calcium, the calcium carbonate biomineral forms around the microbial cell [115]. Here, the present calcium cations are attached to the negatively charged microbial cell surface, which further acts as nucleation of mineral. The presence of heavy metals or radionuclide elements such as Pb, Cr, Ni, and Sr similar to the ion radius of Ca^2+^ can be precipitated or coprecipitated in to the carbonate crystal by substituting Ca^2+^ or by entering in the intervening space of the calcium carbonate crystal [18,116,117]. With this, the heavy metals get immobilized within the crystal structure and gives a suitable solution of remediation [68,87,118–120].

Various research studies have been carried out to immobilize the heavy metals and radionuclides via the ureolysis-driven MICP technique [14, 121, 122, 68, 123], for example, remediation of [124,125] 99.95% of cadmium removal by *Lysinibacillus sphaericus* and by an indigenous ureolytic bacterium, i.e., *Exiguobacterium undae* [126,127], and 97% of copper removal by *Kocuria flava* [126, 54]. Pertaining to copper immobilization, studies have proven that *Sporosarcina koreensis* showed a higher rate of precipitation compared to *S. pasteurii* [128]. The other such examples are remediation of Cr polluted soil by *Bacillus cereus* [122,123,129] and 100% remediation of lead-contaminated soil by different ureolytic bacteria [130; 124, 128]. MICP studies have shown that it is possible to immobilize the radionuclide strontium by coprecipitating it in the calcite lattice via Sr-resistant extremophilic ureolytic bacteria [68,124,131]. Not only single pure ureolytic culture but also MICP bioremediation has been successfully applied in a soil contaminated with Pb, Cd, and Cu by introducing ureolytic bacterial mixtures [121]. 89 to 100% of freely available divalent toxic elements can form insoluble carbonates via the ureolysis-driven MICP remediation technique [132, , 128, 3, 7, 18].

Although these studies have been conducted in laboratory, implementing them under real field conditions has the issue of the ammonia and ammonium by-product generation. Few theoretical studies have shown that the generated ammonia increases the availability of the contaminant via exchanging the sorbed radionuclides or metals with the subsurface in porous media. The increase in availability of pollutant further accelerates the remediation process. However, no experimental studies or proven data are offered on this theoretical concept [87, 118, 120]. It concludes that the ureolysis process not only remediates the polluted soil but also displaces it with another contaminant, i.e., ammonia, which has adverse environmental effects [133]. Also, the aerobic nature of ureolytic bacteria hinders the microbial and biomineralization activity on the subsurface i.e., low or no oxygen conditions [19].

Denitrification-based MICP is an effective and eco-friendly metabolic pathway for soil remediation in terms of (i) inert and nontoxic byproduct, nitrogen gas; (ii) less substrate and low nitrate concentration; (iii) the facultative anaerobic nature of denitrifying bacteria; (iv) highly ubiquitous, (10 to 15% of microbes in soil, sediment and water); (V) high mineral yield and (vi) 100% utilization of the electron donor possibility in biostimulation [66,104,134,135]. It paves the way for investigating and evaluating the feasibility of denitrification-based MICP for remediation purposes under different field conditions.

### 4.3 Restoration of the heritage structure

Architectural structures, limestone monuments and other heritage building have their own social and historical value for the country and world. The surface and entire structures are deteriorated due to various physical, chemical and/or biological weathering, which is a social concern [136]. The deterioration or degradation further has a negative impact such as increasing the material porosity, lessening the mechanical properties, discoloring, inclination, etc. [137]. Different conservation and restoration strategies have been applied to mitigate the irreversible damage or degradation [43,137]. However, conventional chemical treatments have drawbacks, i.e., requirement of high maintenance, continuing the internal degradation without surface damage, temporary applicable or not durable, and generation of noxious compounds, among others [138,139].

Biomineralization proposed an alternative methodology for the restoration of these buildings and monuments by precipitating a layer of biomineral crystals [140–144]. The biological carbonate seals the microcracks, reduces the water absorption, and inhibits the water flow into the stone by decreasing the porosity, which result in a more consolidated structure [145–149]. The carbonate crystals are more resistant pertaining to stress and chemical reactions than the original stone and provide an appropriate eco-friendly and inexpensive solution of restoration [18,43].

Different ureolytic bacteria such as *B. subtillis, B. sphaericus*, and *B. lentus* have been applied on damaged limestone monuments [150,151]. Two field restoration implementations on the Saint Medard Church’s tower and Santa Maria church in Italy have proven that the MICP treatment is a feasibility solution, which do not affect the esthetic appearance of heritage structures [152]. In 2000, the first study by Castanier et al. isolated a denitrifying bacterium, i.e., *B. cereus,* and started utilizing it to restore the historic buildings. The different strains of denitrifying bacteria such as *P. stutzeri* and *P. aeruginosa* have been utilized to remove the chemicals from the wall paginating and stones under bench-scale laboratory conditions [153,154]. In 2013, biorestoration of ornamental stone was carried out by the isolated *P. chlororaphis* [155].

A few studies have been carried out to implement the MICP treatment on restoration of heritage buildings [152]. A detailed investigation on the nature of mineral precipitated, its effect on the restoration of stones and building, and the durability of the MICP application under adverse condition needs to be carried out to implement under in situ conditions.

### 4.4 Self-healing concrete

Concrete is a most widely used building material for various construction purposes due to its high strength and low permeability. It is made of a certain ratio of cement, coarse particles and water. The average life of concrete is nearly 50 years and after that, it slowly deteriorates due to weathering and form cracks [156,157]. Furthermore, the durability decreases due to shrinkage, rapid loss of water sun light, acid rain and ingress of various chemicals in the crack [158, 159, 160].

The formed crack can be sealed by the precipitation of the carbonate crystal inside and outside the surface of concrete via biomineralization or MICP technique. This is known as the self-healing property of concrete or bioconcrete, which improves the mechanical and durability properties of concrete structures [8,161]. The low maintenance and repair cost and no CO_2_ emissions made the bioconcrete make it one of the most eco-friendly technologies to remediate the concrete structure [8]. Several studies have been carried out for self-healing the cracks of concrete by utilizing various microbes such as *B. pseudofirmus, B. subtilis, B. alkalinitrilicus, P. aeruginosa*, etc. during either ureolysis or denitrification-based mineralization [161–163, 164]. Although microbial ureolysis gave successful results for bioconcrete, it has several drawbacks, i.e., the microbial activity and mineralization hindered inside the crack with the absence of oxygen and the generation of ammonia as a toxic byproduct [22]. Various studies have been carried out to protect the microbial cell inthe harsh concrete environment by injecting nutrient sources (lactate, spores enriched with urea, yeast extract) and different materials, i.e., polyurethane, ceramics, cementitious material, calcium sulphoaluminate cement, various nanoparticles, etc. for successfully healing the crack via the microbial ureolysis process [157,162,165–173]. However, utilizing these strategies involved huge cost for crack healing and improving mechanical properties [8,174].To overcome this, microbial denitrification has been applied for enhancing the mechanical and durability properties of concrete [22,164,175].

Several studies have been conducted for utilizing different nitrifying strains such as *Pseudomonas aeruginosa* and *Diaphorobacter nitroreducens* protected within either granular activated carbon particles or expanded and can heal the cracks of 400–470 μm width upon 7 weeks [164,176–178]. Furthermore, the same study has been extended by utilizing non-axenic cultures using a special granulated bacterial culture called activated compact denitrifying core. These biogranules are more effective in healing the crack of 500 μm wide and inhibiting the steel corrosion and the microbes can survive in high cement (in 3% w/w cement incorporation dose) and mortar [176,179,180]. Interestingly, it was shown that these biogranules can self-heal the 400 μm wide cracks during wet-dry cycles and old specimens can also be repaired with a similar mechanism [135,181]. In the mortar specimen, a 10 mm calcite thick layer has been precipitated after 4 weeks, which assist in reducing the permeability by 83% by a reduction of 6% crack volume [182]. Another advantage of utilizing the microbial denitrification over ureolysis is the inhibition of steel corrosion by the production of NO_2_ (produced as intermediate waste during denitrification), a well-known anodic corrosion inhibitor [182]. The study shows that although similar crack repair occurred in both ureolysis and denitrification, only denitrification-based self-healing concrete can protect the steel bar against corrosion and hence can be suitable for structures exposed to marine (aggressive ions and wet dry cycle). Although a promising study was carried out for self-healing concrete via denitrification, kinetics of microbial growth, and their metabolic and enzymatic activities, the fundamental mechanism of self-healing via microbial species in a concrete environment needs to be identified in detail to appreciate the phenomenon under real in situ conditions. However, the experimental study concludes that MICP through denitrification is a promising technique for a multifunctional self-healing bioconcrete.

## 5. Challenges in denitrification for *in situ* application and perspectives

This manuscript presents an overview of the successful denitrification-based MICP process in laboratory experiment. But implementing it under real field conditions on a commercial scale faces several biotic and abiotic challenges, discussed in this section.

### 5.1 Types of microbes used

The first challenge of this microbial process is the selection of the suitable microbes pertaining to the application. Unlike different ureolytic bacteria that are already recognized as the most suitable microbe for carbonate precipitation, no standard or specific denitrifying bacteria are widely accepted for an efficient denitrification-based MICP. Initial works should be carried out to isolate, select some suitable organism or modify the microbes for higher enzymatic activity, denitrification abilities and precipitation yield. Selection and classification of suitable denitrifying bacteria by using phylogenetic classification is not a practical tool in the ecological design of MICP because this classification system does not consider the physiological characteristics pertaining to its denitrifying capacity. Hence, a classification system is highly required based on different criteria such as growth environment, rate of enzymatic activity, nitrate and organic matter requirement, energy requirement, rate of precipitation, etc. [183].

Although some of the studies repaired the crack of concrete or enhanced soil properties via the biostimulated denitrification process, isolation and characterization of the microbes involved in the process has not been performed [22]. Hence, the involvement and efficiency of the microbial species is still in doubt. It concludes that identification of the mixed or pure culture of microbes, biochemical analysis, comprehending their growth kinetics, enzymatic activity, and mineral precipitation mechanism are highly required for the real-field implementation of the MICP process. Few studies have investigated the direct relation between the denitrification and biomineral formation and precipitation around the microbial cells during denitrification [135]. It is also essential to understand the effect of the type of microbes on the rate of modification during MICP treatment via denitrification.

### 5.2 End product of the denitrification process

MICP via denitrification is a multistep metabolic reaction with the involvement of different microbes and enzymatic activity. In the laboratory, the whole multistep denitrification process was carried out under optimum and controlled conditions. However, there is a high chance of the partial or incomplete occurrence of denitrification reactions, which leads to the generation of toxic intermediate products such as nitrite, nitric oxide, and nitrous oxide [103,104]. In nature, more than 50% of NO_x_ compounds are generated by biologically denitrification and/or nitrification on a global scale. These intermediate products have an undesirable effect on human health, agriculture, aquatic life and ecosystem.

Unlike ammonia, it is not bounded in soil and hence easily transported to larger distances and harmful to the society. A recent study has utilized the intermediate nitrite for microbial self-healing concrete applications [176,182]. However, different strategies have to be planned to complete the denitrification process for biomineral formation and MICP modification. Not only the environment but also the strategies are essential for investigating the effect of this intermediate waste on the growth and enzymatic activity of denitrifying microbes. The generation of the final end product, i.e., nitrogen, its further utilization for soil improvement, and its effect on the environment need to be studied further.

Utilizing the nitrogen gas for engineering application such as liquefaction mitigation also faces potential challenges, i.e., control of the generation, persistence and distribution of the gas over the treatment zone, etc. For example, studies have concluded that the gas bubbles are not stable in the long sand column and flow out from the intended zone in low confinement [94,96]. Also, high uncontrolled production of gas initiates cracks in soil structures for low confinement conditions [104].

### 5.3 Effect of prevailing chemical and environmental conditions

In laboratory experiments, the abiotic factors such as pH, temperature, pressure, concentration of nutrients, salinity and biotic factors, i.e., sterile condition without any other microbes, are controlled to investigate the MICP modification technique via denitrification. But in contrast to the laboratory, these parameters vary significantly and cannot be controlled in real field applications such as for soil improvement, restoration of building materials, durability of concrete, etc [184]. Also, these mentioned factors affect the survival, growth, metabolic and enzymatic activity of denitrifying microbes, generation and transportation of the microbes and denitrification reaction products, rate of biomineral production and nature of biomineral significantly [82,164,185,186].

Hence, a detailed understanding and optimization study are needed focusing on the influence of various biotic and abiotic factors on denitrification, mineral formation and nature of biomineral by considering the complexity of natural soils, groundwater, concrete and other building materials. In this regard, a biogeochemical model without any flow condition has been developed to simulate the process of MICP via denitrification [187–189]. However, a suitable mathematical modeling, designing appropriate monitoring systems will be helpful to investigate and quantify the effect of these factors on the rate of enhancement and for successfully developing the MICP technique in the field.

### 5.4 Sustainability

This article shows the successful development of the denitrification-based MICP process and the upcoming challenges to implement it in a variety of engineering applications. Owing to their abundance in subsurface soils and groundwater, denitrification-based MICP can be employed for waste management in terms of in situ remediation of metal contaminants, mitigating soil pollution and for sustainable building materials in terms of self-healing concrete, brick, strengthening various geomaterials and modifying the unsuitable soil for construction. The application of the MICP process in different fields of engineering promotes the sustainable land use planning and management and sustainable construction industry activities. These both come under one of the goals ‘Sustainable cities and communities’ out of seventeen goals of Sustainable Development of Department of Economic and Social Affairs, United Nation.

The three pillars of sustainability are durability, feasibility in terms of cost and environmental viability. Pertaining to durability, it is essential to evaluate the stability of MICP-based remediation or reinforcement over a time span and its durability under adverse conditions. None of the studies have been conducted to comprehend the long-term effect of denitrification-based MICP application. Hence, the long-term durability of denitrification-based MICP demands a detailed examination by considering the phase transformation of biomineral formed after the treatment period, effect of the adverse conditions such as acid or alkaline attack, wet-dry and frost-heave condition on the nature of biomineral formed and MICP improvement.

As per 183, $2 to $72 cost is required for the raw material in chemical grouting, whereas only $0.5 to $9.0 is needed in MICP-based biogrouting for one m^3^ of soil treatment. In this process, utilizing various waste for microbial nutrient and cementation reagents makes not only the MICP process cost-effective but also environmental friendly [190, 191, 25, 172, 192–194, 5, 160]. Also, the ubiquitous nature of denitrifying microbes assists in implementing the MICP technique via biostimulation and makes the process more cost-effective compared to other augmentation-based MICP techniques. However, a cost analysis pertaining to microbes, chemicals, advance techniques and energy involved in the denitrification process should be done to analyze its feasibility over other chemical or physical treatment processes in large-scale application.

Pertaining to environmental viability, Ref 195 had compared the environmental impacts for designing a concrete block pavement by cement and MICP-based technology. The study concludes that MICP treatment has 19% more environmental and cost benefits compared to the cement-based concrete pavers. However, making sub-base and sub-grade via MICP treatment is neither environmental friendly nor cost-effective pertaining to energy consumption and CO_2_ emission. The study also infers that alternative solution can be explored such as waste of milk industry, i.e., lactose mother liquor for microbial nutrient can lower the cost and energy emission in MICP treatment [172].

Since the MICP technique is a natural biological process, it should be more environmental friendly compared to other chemical treatment. However, a life cycle assessment needs to be carried out to analyze the environmental impact of the denitrification-based MICP application pertaining to energy required, waste production, and effect to the environment after long-term installation such as leachability. The sustainability analysis can assist in implementing the denitrification process more efficiently on a commercial scale.

Apart from all the mentioned factors, modeling the biogeochemical process can help to predict and comprehend the MICP behavior in a better way [196]. In this regard, some of the studies have proposed different empirical relationships to predict the strength and stiffness of biomodified soil as a function of quantity of biomineral formed, shear wave velocity, soil type, etc. [197–200]. Although a lot of influencing factors are not included in the empirical equation, it provides a future path to develop a better model. Although different DEM modeling approaches by using various bond models and constitutive modeling have been modeled to analyze the constitutive and microscopic behavior of cemented soil treated by the chemical process, few models have been developed for MICP-treated cemented soils. For example, Ref 201 has modified an existing constitutive model by adding a cementation parameter to analyze the degree of cementation and its effect on soil reinforcement. Also, in 2018, Gai and Sanchez analyzed the MICP-based soil reinforcement by developing an elastoplastic constitutive model. In the DEM modeling, a bond model is developed to represent the contact between the soil particles and biomineral formed during MICP [195,202–205]. The various bond models utilized in MICP-based DEM modeling are parallel bond model, cement ring bond model and cohesive bond model [195]. But these modeling studies are limited to very few real-field scenarios. In the future, developing an appropriate biogeochemical modeling by introducing all the influencing factors will help to comprehend the MICP mechanism in a better way.

## 6. Conclusions

Overall, this article demonstrates that microbial induced precipitation via denitrification has great potential to resolve a wide range of building material problems such as ground modification, mitigating the liquefaction and soil pollution, improving the durability and engineering properties of concrete, historic buildings, monuments, etc. under aerobic and anaerobic conditions. Not only this but also the studies are extended for use in different chemical, environmental and biomedical-related science and applications. This biogeochemical process is a revolution in civil engineering where the geomaterials are no more considered as inert abiotic engineering materials. However, various challenges are needed to be addressed before utilizing it in a large-scale application or as a commercial purpose. It is an interdisciplinary research, which needs an involvement of a large number of researchers and industrialisst from different backgrounds of microbiology, biochemistry, geology, and geotechnical engineering.
